# Uncommon Systemic Manifestation of Group A Beta-Hemolytic Streptococcus in a Middle-Aged Woman: A Case Report

**DOI:** 10.7759/cureus.78863

**Published:** 2025-02-11

**Authors:** Tetsuya Akaishi

**Affiliations:** 1 Department of Education and Support for Regional Medicine, Tohoku University Hospital, Sendai, JPN

**Keywords:** afebrile, anti-streptolysin-o, group a beta-hemolytic streptococcus, rheumatic fever, staphylococcus pyogenes

## Abstract

Group A beta-hemolytic *Streptococcus* (GABHS) is a gram-positive type of bacteria, typically causing high fever and painful pharyngitis. The bacteria may sometimes trigger widespread skin rash in children, but systemic conditions other than sore throat are rare in adult patients with the infection. A 57-year-old woman visited our hospital with a sore throat, pruritic skin rash in the body trunk, painful oral ulcer, swollen lips, arthralgia, and swollen left wrist joint with pain. She had a recent similar clinical episode approximately four weeks before the hospital visit, which was alleviated with oral loxoprofen. The swelling of the left wrist had migrated from the left elbow area in the preceding four weeks. The patient had similar symptoms about 30 years ago, which were diagnosed with GABHS infection and successfully treated with oral antibiotics. Based on this past similar clinical episode, the patient was evaluated by the GABHS rapid antigen detection tests, which revealed a positive result. The blood test data showed normal white blood cell (WBC) count and C-reactive protein (CRP) level. She was free of any serum antibodies associated with autoimmune connective tissue diseases or syphilis. Deciding the diagnosis was difficult, but based on her past similar clinical episode in her 20s and systemic conditions resembling rheumatic fever, a diagnosis of GABHS-related uncommon systemic response was made. The patient was treated with clarithromycin (200 mg, twice a day) for 10 days and amoxicillin (250 mg, three times a day) for an additional seven days, and all symptoms resolved. Four weeks after the first hospital visit, the serum anti-streptolysin-O (ASO) level was normal at 135 IU/ml. An echocardiogram revealed an anterior mitral leaflet calcification with a mild level of mitral regurgitation. The present case indicated the importance of considering GABHS infection in adults with strong sore throat, widespread skin rash, arthralgia, and swollen joints with uncertain causes, even when the patient is afebrile with normal WBC count, CRP level, and ASO titer.

## Introduction

*Streptococcus pyogenes*, also known as group A beta-hemolytic *Streptococcus* (GABHS), is a gram-positive type of bacteria that can cause upper respiratory tract-related symptoms both in children and adults [1,2]. This microorganism typically causes acute tonsillitis/pharyngitis with tender anterior cervical adenopathy, red throat, and swollen tonsils with exudates, which are incorporated in the Centor Score for diagnosing GABHS-related pharyngitis [3]. In daily clinical settings, tonsillar swelling and exudates are often absent, although they are indicative of GABHS infection [4]. The bacteria may also cause erysipelas when the dermis layer of the skin is infected [5]. The exotoxins of GABHS (e.g., cytolytic streptolysins, erythrogenic/pyrogenic toxins) may further trigger strong inflammatory responses. These exotoxins play important roles in developing non-respiratory systemic conditions represented by rheumatic and scarlet fever, typically presenting high fever and various types of widespread skin rashes [6,7]. The wide variety of delayed non-respiratory systemic symptoms caused by GABHS infection and the exotoxins may sometimes make the diagnosis difficult, especially when either or both high fever and strong sore throat are absent. This case report presents uncommon systemic manifestations, including normal body temperature, pale skin rash, and swollen wrist joint in a middle-aged woman.

## Case presentation

**Figure 1:**
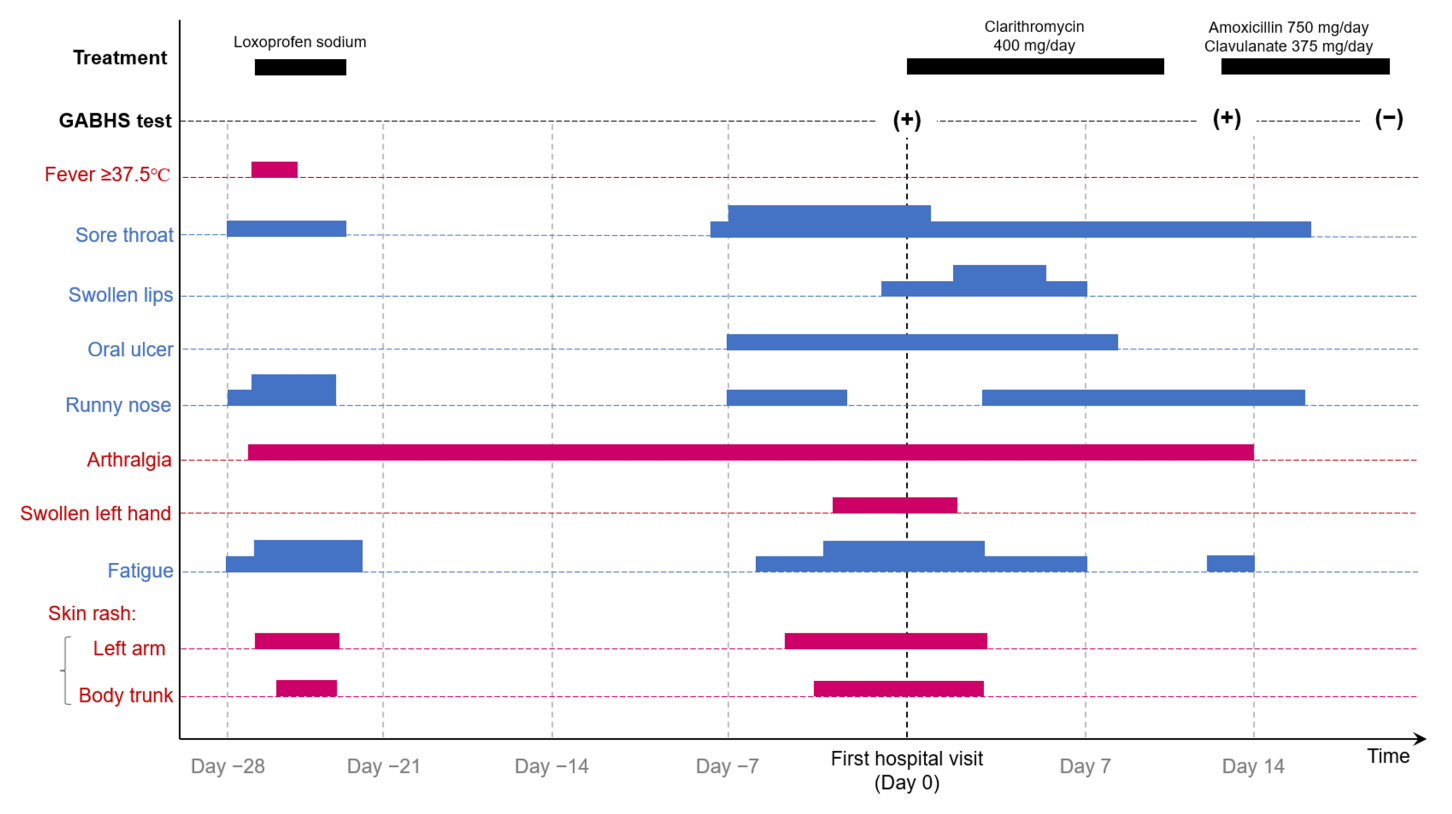
Clinical course of the 57-year-old woman with GABHS infection Her clinical episode began four weeks before the first hospital visit with fever, sore throat, runny nose, arthralgia, fatigue, and widespread skin rash in the body trunk and left arm. These symptoms, except for the arthralgia, once subsided with oral loxoprofen sodium. Two weeks after this resolution, she noticed a recurrence of these symptoms. Because she experienced similar symptoms with the diagnosis of GABHS infection about 30 years before, she decided to visit our hospital to undergo the GABHS antigen rapid testing. GABHS: Group A beta-hemolytic *Streptococcus*

A 57-year-old woman with no notable past medical history or medication history visited our hospital for the first time with pruritic systemic skin rash, painful oral ulcer, swollen lips with a burning sensation, and painful swelling of the left wrist joint. The patient experienced miscellaneous systemic symptoms with uncertain causes, such as fever, sore throat, runny nose, fatigue, systemic arthralgia, pruritic skin rash in her body trunk and left arm, and pruritic blister formation in her left flexor side of the elbow (two blisters with the sizes of 5-10 mm), from approximately four weeks before the first hospital visit. She took oral loxoprofen sodium, and these symptoms, except for the arthralgia, gradually subsided within the next 3-4 days. The two pruritic blisters in her left elbow were gradually exchanged with pruritic rashes in the left forearm and wrist, which she expressed as “migratory” skin rash and pruritus. Eight days before her first hospital visit, she experienced the recurrence of sore throat, soon followed by those of the other symptoms. Upon this recurrence, she recalled her previous clinical episode at the age of 28 years, when she had prolonged similar symptoms with high fever ≥38.0℃ for several weeks and finally was diagnosed with GABHS infection by a pediatrician. The pediatrician advised her to receive a GABHS antigen testing when she develops similar symptoms in the future. Based on this past experience in her 20s, she considered the possibility of GABHS infection for the present manifestations and visited our hospital to receive antigen testing. She reported no other symptoms suggestive of rheumatic fever, such as thoracic (e.g., chest pain, palpitation), abdominal (e.g., abdominal pain, bloody stool), or neurological symptoms (e.g., chorea). Her clinical course with observed conditions and treatments is shown in Figure 1.

For reference, information on past clinical episodes from about 30 years before was obtained from her. At 28 years old, she developed a prolonged high fever ≥38.0℃ lasting for more than 4 weeks and a pruritic systemic skin rash that started in her legs and spread to the body trunk. Lip swelling was absent. She first visited an internal medicine physician and dermatologist, both of whom gave her a diagnosis of severe urticaria, but the administered medications were not effective. She finally consulted a pediatrician, who finally gave her the diagnosis of GABHS infection based on antigen testing. The pediatrician told her that the observed symptoms of the disease are quite rare, especially in adults. He advised her to undergo GABHS antigen testing if she develops similar symptoms in the future. The pediatrician prescribed oral antibiotics and topical corticosteroids, both of which were effective, and the fever swiftly receded.

**Figure 2:**
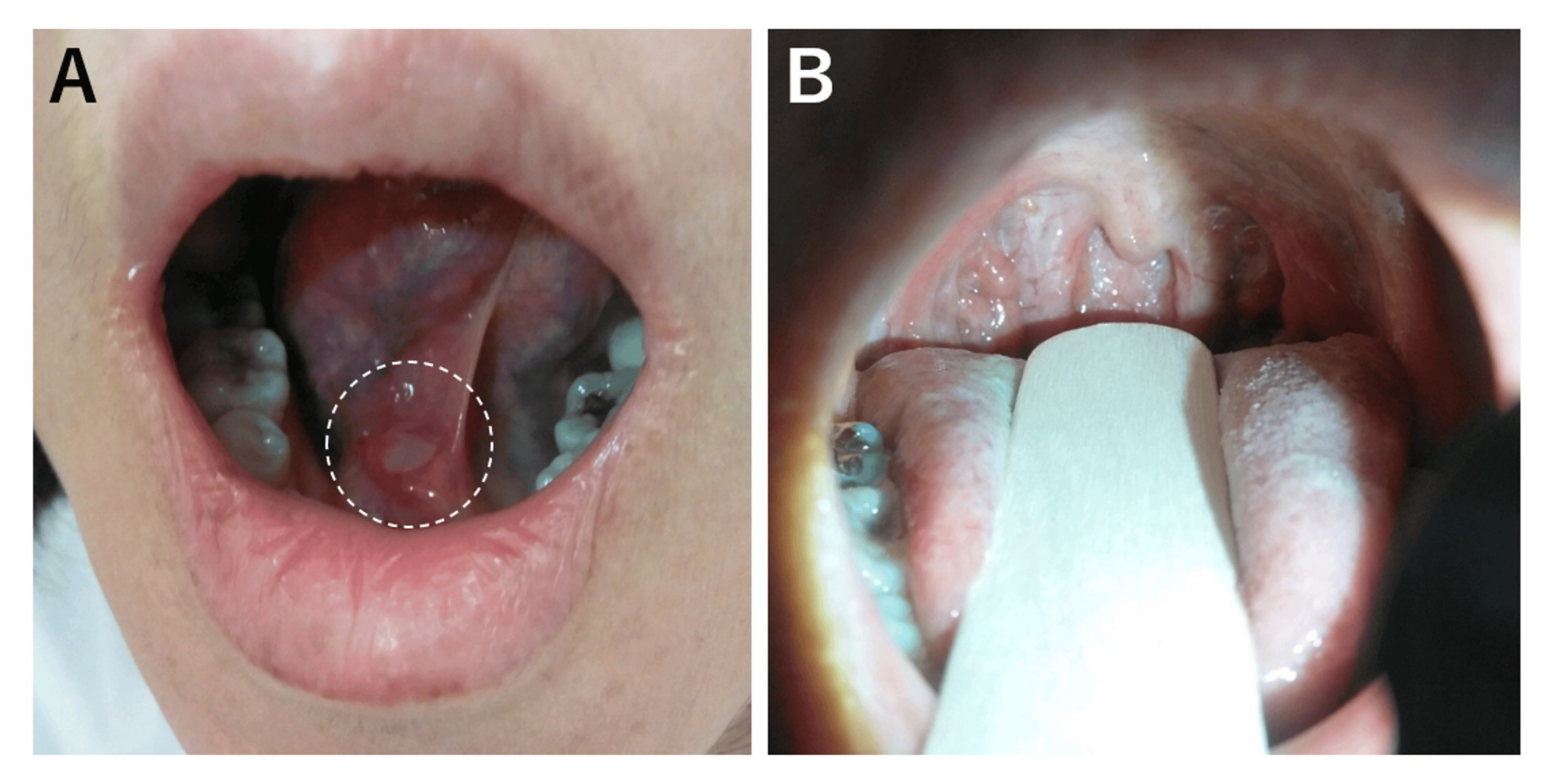
Oral ulcer and oropharyngeal finding at the first hospital visit (A) A painful oral ulcer below the tongue (white circle) was present. (B) The oropharyngeal mucosa was slightly reddish, but there was no tonsillar swelling or exudates.

**Figure 3:**
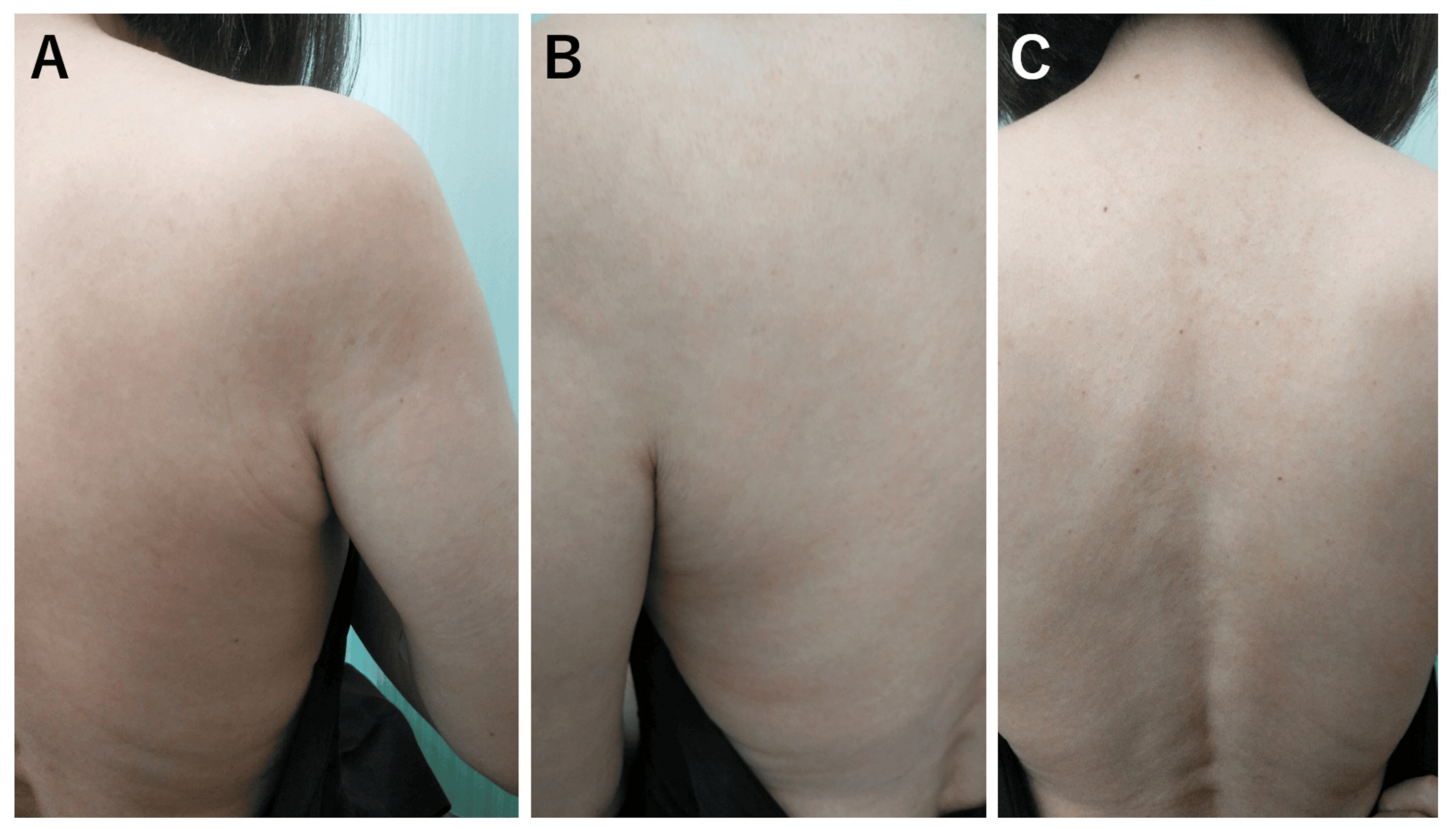
Pruritic skin rash in the body trunk at the first hospital visit The pruritic skin rash in her right upper back (A), left upper back (B), and middle upper back (C) are presented. These rashes or itchiness did not subside with intravenous histamine H1 receptor blockers.

**Figure 4:**
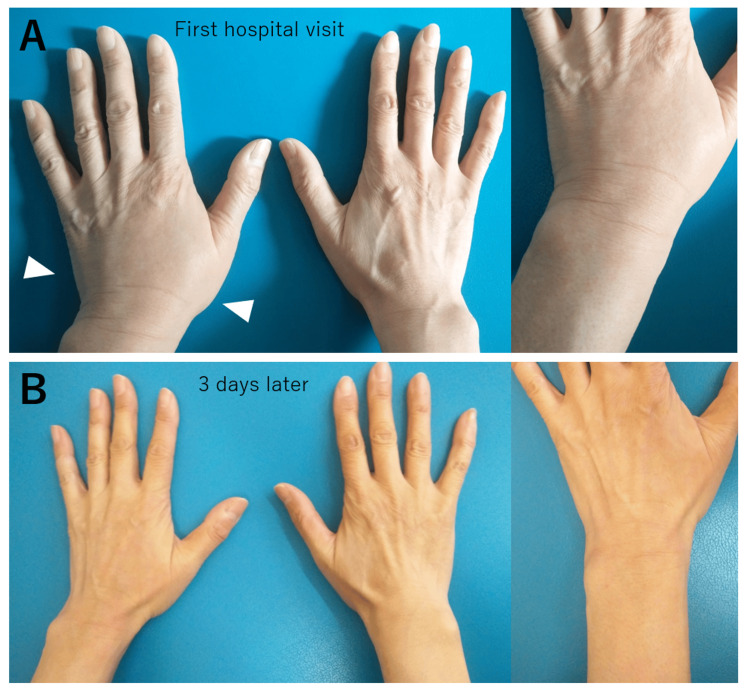
Swollen left wrist joint at the first hospital visit and resolved findings 3 days later (A) At the first hospital visit, her left hand and wrist were swollen with slight pain (white arrowheads). (B) These findings were swiftly resolved by 3 days after starting the oral antibiotic drug.

**Figure 5:**
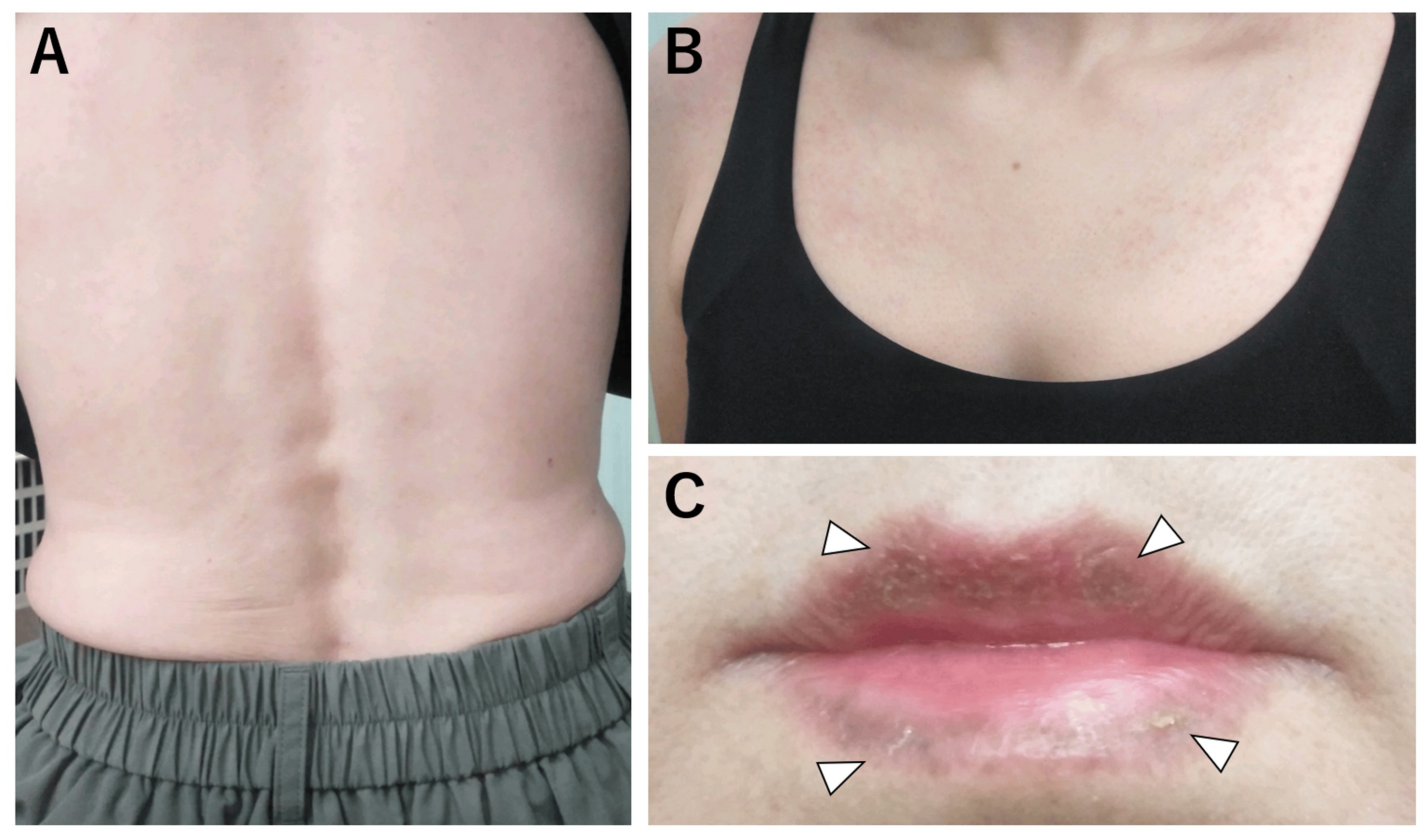
Resolving skin rashes and lip swelling at 3 days after starting oral antibiotics Resolving skin rashes in her lower back (A) and anterior chest (B) after 3 days from starting oral antibiotics are shown. (C) Lip swelling was also resolving with brownish crust formation (white arrowheads).

Approximately 30 years later, after this past episode, she visited our hospital with similar symptoms at 57 years old. She had a fever lasting for several days, about four weeks before the hospital visit, but she was afebrile at the hospital visit at 36.0℃. The blood oxygen saturation level was 99% on room air. She had a painful oral ulcer below her tongue (Figure 2A), but her oropharyngeal findings were not so remarkable (Figure 2B). Her pruritic skin rash in the body trunk is shown in Figure 3. The rashes and itchiness did not resolve at all with intravenously administered d-chlorpheniramine maleate (5 mg). She also had swelling in her left hand and wrist joint, and the swollen area was slightly painful (Figure 4A). Because the patient reported that these symptoms are quite similar to those she experienced 30 years ago, the GABHS rapid antigen test with QuickNaviTM-Strep A2 (Denka Co., Ltd, Tokyo, Japan) was performed, the result of which was positive. Her blood test results at the first hospital visit are shown in the middle column of Table 1, which revealed no elevated white blood cell (WBC) count and serum C-reactive protein (CRP) level. Based on her past clinical episode and the positive result of the GABHS antigen test, uncommon systemic manifestation related to GABHS infection was considered. At this season, amoxicillin was in short supply all across Japan, which was also unavailable at our hospital, and oral antibiotics with clarithromycin (200 mg, twice a day) for 10 days were started. After starting the antibiotics, sore throat and skin rash swiftly started to coalesce and resolve within several days (Figure 5A, 5B). The patient also reported that topical corticosteroid (hydrocortisone butyrate) was also effective in relieving the itchy sensation. The patient reported an exacerbation of lip swelling with a burning sensation after the first hospital visit, but it also gradually resolved with a brownish crust formation (Figure 5C). Because her clinical symptoms were atypical for GABHS infection in adults, GABHS antigen testing was repeated at her own expense 13 days after the first hospital visit, which revealed a positive result again. Considering the remaining sore throat, additional oral antibiotics were decided to be administered. This time, amoxicillin was available, and amoxicillin (250 mg, three times a day)-clavulanate potassium (125 mg, three times a day) for seven days was started. By the fifth day of starting the additional antibiotics, all symptoms were totally resolved, and the reperformed GABHS antigen testing 21 days after the first hospital visit revealed a negative result at last. After the treatments, the urine test was normal, with negative urine occult blood or urine protein.

**Table 1:** Blood test results at the first hospital visit and 13 days later after treatment ALT: Alanine aminotransferase; AST: Aspartate transaminase; BUN: Blood urea nitrogen

	First hospital visit	13 days later
White blood cell count [/μl]	4,400	3,900
Hemoglobin level [g/dl]	14.3	14.1
Platelet count [10^4^/μl]	15.3	17.0
AST [U/l]	21	22
ALT [U/l]	17	14
BUN [mg/dl]	15.8	20.2
Creatinine [mg/dl]	0.55	0.62
C-reactive protein [mg/dl]	< 0.10	0.19

**Figure 6:**
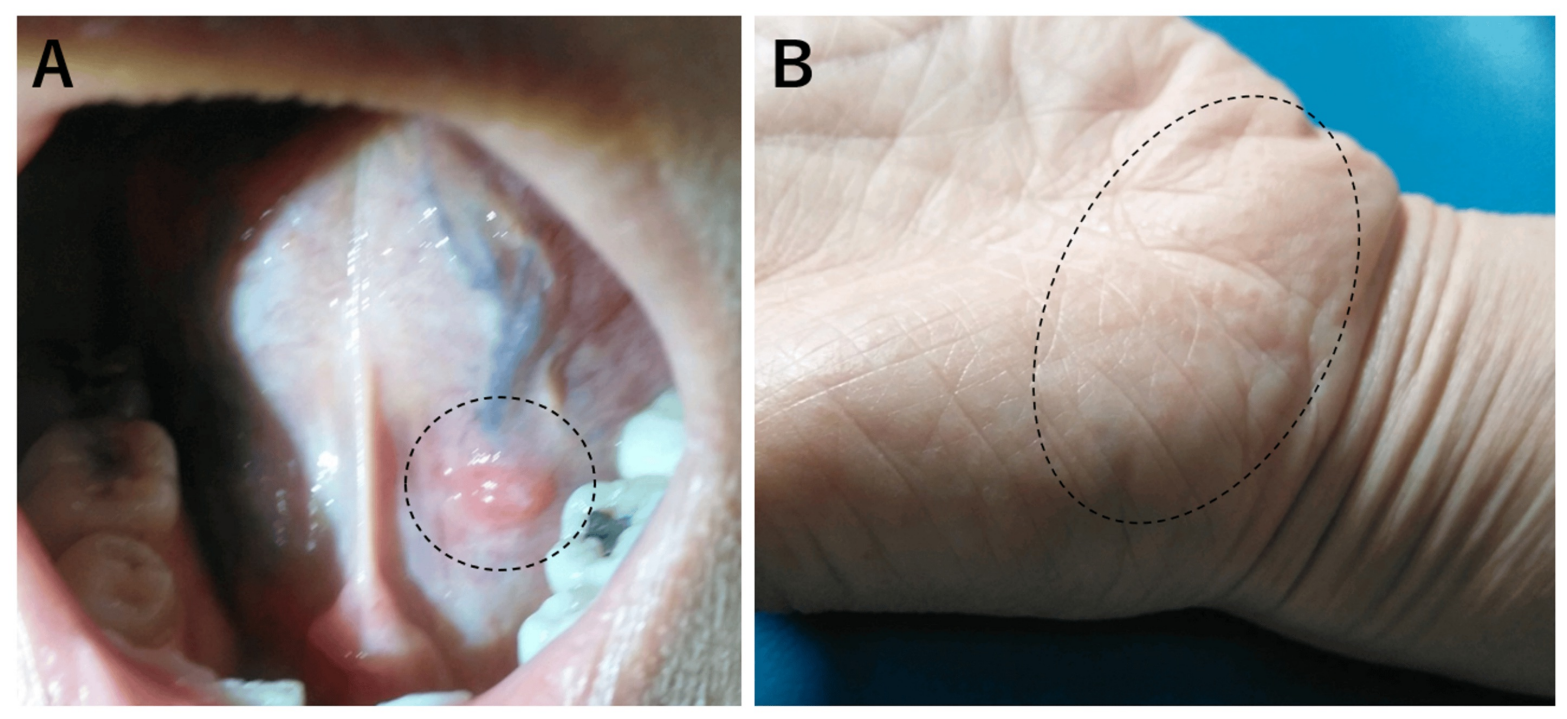
Another oral ulcer and pruritic small nodules in the left hand after one month from the first hospital visit (A) Another painful oral ulcer on the other side of the lingual frenulum below the tongue (black circle) was present one month after the first hospital visit. (B) Pruritic small skin nodules with rough, lumpy surfaces were left behind after the swelling of the left wrist joint receded.

**Figure 7:**
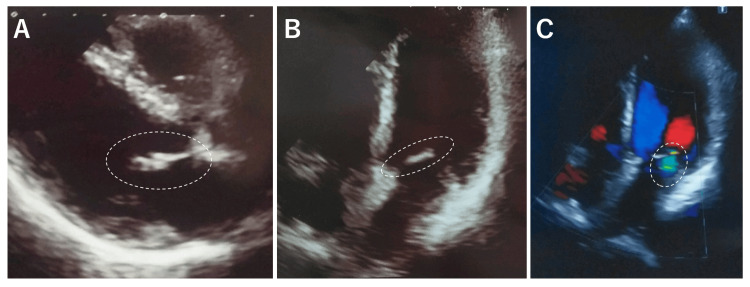
Echocardiogram showing possible anterior mitral leaflet calcification with mild mitral regurgitation at 4 weeks after the first hospital visit (A) Long-axis view suggested the presence of anterior mitral leaflet calcification (white ellipse). (B) Four-chamber view. (C) Four-chamber view in color Doppler echocardiography showing the presence of mild mitral regurgitation (white ellipse).

To exclude the possible diagnosis of rheumatic fever and the risk of cardiovascular events in the future, she underwent a comprehensive cardiovascular follow-up one month later. This time, she had another painful oral ulcer on the other side of the lingual frenulum below the tongue (Figure 6A). The crust formation of her lips had been completely resolved, and her lips appeared normal. She reported the presence of pruritic, painless small nodules with a rough, lumpy surface in her left hand, which became clear after the withdrawal of left wrist swelling (Figure 6B). A cardiac murmur was absent, but the electrocardiogram suggested the presence of anterior mitral leaflet calcification with mild mitral regurgitation (Figure 7). Serum anti-streptolysin-O (ASO) titer was normal at 135 IU/ml (normal range: <240 IU/ml). The serum antinuclear antibody titer was mildly positive at 1:160. However, any of the rheumatoid factor, anti-double-stranded DNA antibody, anti-SSA and anti-SSB antibody, MPO- and PR3-ANCA were all normal. Furthermore, the syphilis-related blood tests (i.e., RPR, TPHA) were normal.

The small nodules on her left palm gradually disappeared in the following several weeks. Because most of the symptoms receded, the serum ASO level was normal, and the pharyngeal GABHS rapid antigen detection tests turned to a negative result, the patient decided not to take long-term antibiotics or corticosteroids. However, as some of the observed symptoms seemingly resemble those seen in patients with rheumatic fever, an echocardiogram is planned to be regularly followed up for several months, and she was also asked to revisit the hospital when she has a recurrence of similar symptoms in the future. Currently, at two months from the first hospital visit, she is free of any symptoms or recurrence without taking any medications.

## Discussion

In this case report, a middle-aged woman with uncommon systemic symptoms related to GABHS infection, such as widespread pruritic skin rash, swollen lips, arthralgia, and a swollen wrist, was presented. At the first hospital visit, she was afebrile with a normal WBC count and CRP level, and making a diagnosis based on the GABHS rapid antigen detection tests was a difficult decision. However, the diagnosis was decided based on her past similar clinical episode with GABHS infection about 30 years ago and her present clinical manifestations resembling rheumatic fever. All symptoms other than a painful oral ulcer at a different site and pruritic skin nodules at the withdrawal site of monoarthritis swiftly receded within two weeks after starting the treatment with oral antibiotics. This successful treatment with oral antibiotics may further support diagnosing GABHS-related systemic response. The patient was free from any cardiovascular symptoms like chest pain or neurological symptoms like chorea. Because most adults with GABHS infection present isolated pharyngitis without other systemic conditions in non-respiratory systems, the observed symptoms were uncommon, and the diagnosis was difficult. GABHS typically provokes strong inflammatory reactions in the human body by producing hemolytic exotoxins, such as streptolysin O and streptolysin S, which are known to potentially provoke apoptosis of some immune cells including macrophages and neutrophils [8], potentially resulting in rheumatic fever in the following several days to weeks. Human immune systems produce neutralizing antibodies, such as ASO, against these virulence factors, but not in all cases with the infection and it may be found weeks or months after the GABHS infection [9]. To be noted, 10-20% of asymptomatic school-aged children are suggested to be colonized with S. pyogenes [10-12]. Most of the asymptomatic people colonized by the bacteria will show normal ASO levels. Therefore, the diagnosis of GABHS infection must be carefully made based on the clinical findings, referencing an elevated serum ASO level in the acute phase of infection. A normal ASO level may decrease the likeliness of GABHS infection, but it does not necessarily rule out the diagnosis.

The overview of her clinical course initially reminded an impression of rheumatic fever. Although rheumatic fever is commonly seen in children aged 5-15, it can also affect adults [13]. Meanwhile, her symptoms did not strictly fulfill the 2015 revision of the Jones criteria for the diagnosis of acute rheumatic fever [14,15]. To be noted here, the absence of fever is not enough to exclude acute rheumatic fever. The presence of fever is only among the minor criteria, and afebrile rheumatic fever is theoretically possible. However, as for the present case, she fulfilled none or one of the major criteria (arthritis) and only one minor criterion (polyarthralgia), making the diagnosis of rheumatic fever based on the current diagnostic criteria unlikely. Together with the normal serum ASO level after the treatment with antibiotics, the patient decided not to start long-term administration of antibiotics or corticosteroids. The patient is planned to be rechecked with an echocardiogram approximately 6-12 months later, not to overlook rheumatic heart disease based on chronic subclinical inflammation [16].

As a limitation of this case report, the ASO titer was not evaluated at the timing of clinical onset, although one month after the first hospital visit after treatment with oral antibiotics, it was normal. Serum ASO titer is not incorporated in the diagnostic criteria of rheumatic fever, but paired data of ASO titer before and after the treatment may offer information supporting the diagnosis of invasive GABHS infection with systemic symptoms and to infer the pathological mechanisms underlying the symptoms in the present case [17].

## Conclusions

A middle-aged woman with uncommon systemic symptoms supposedly related to GABHS infection, including pruritic skin rash, swollen wrist joint with pain, swollen lips, and painful oral ulcer, was introduced. At the first hospital visit at four weeks after the clinical onset, she was afebrile, and the blood test data revealed normal WBC count and CRP level. The patient was treated with oral antibiotics for two weeks, and all symptoms were resolved shortly thereafter. The present case suggested the importance of considering GABHS infection in patients with a sore throat, systemic skin rash, and arthritis with uncertain causes, even when the patient is afebrile and the WBC count and CRP level are normal.
